# Output Characteristics and Circuit Modeling of Wiegand Sensor

**DOI:** 10.3390/s19132991

**Published:** 2019-07-07

**Authors:** Xiaoya Sun, Tsutomu Yamada, Yasushi Takemura

**Affiliations:** Electrical and Computer Engineering, Yokohama National University, Yokohama 240-8501, Japan

**Keywords:** Wiegand sensor, FeCoV wire, Wiegand pulse, energy harvesting

## Abstract

A fast magnetization reversal in a twisted FeCoV wire induces a pulse voltage in a pick-up coil wound around a wire. The Wiegand sensor is composed of this magnetic wire and the pick-up coil. As the output pulse voltage does not depend on a changing ratio of the applied magnetic field to switch the magnetization of the wire, the Wiegand sensor is used for to perform rotation and other detections. Recently, the Wiegand sensor has attracted significant attention as a power supply for battery-less operation of electric devices and for energy harvesting. In this study, we propose a concept of obtaining an intrinsic pulse voltage from the Wiegand sensor as its power source, and demonstrate its effectiveness in circuit simulation. The equivalent circuit for the Wiegand sensor is expressed by the intrinsic pulse voltage, internal resistance, and inductance of the pick-up coil. This voltage as a power source and circuit parameters are determined by MATLAB/Simulink simulation. The output voltage calculated using the equivalent circuit of the Wiegand sensor agrees with the experimentally measured results.

## 1. Introduction

The power supply for electric devices connected to the internet is significant to the realization of the Internet of Things. It is not often easy to use power cables based on the location of devices and the high cost that that incurs. Using a battery may prevent the need for power cables; however, the battery needs to be replaced after a certain period of time. Currently, energy harvesting is attracting considerable attention as a technical method for the battery-less operation of devices [1]. In this research, the Wiegand sensor [2,3] is studied as a power generation element for energy harvesting. This sensor has the advantage of output pulse voltage which is independent of the changing rate of the externally applied magnetic field [4].

When the magnetization of a magnetically-bistable wire is switched, a pulse voltage is induced in a pick-up coil wound around the wire. This is known as the Wiegand effect [2,3]. A twisted FeCoV wire, the so-called Wiegand wire, has been used as one of the optimum magnetic wires yielding this phenomenon [5,6]. The fast magnetization reversal of this wire is accompanied with a large Barkhausen jump [7,8]. The Wiegand sensor is comprised of a magnetic wire, normally a twisted FeCoV wire, and a pick-up coil wound around it. Owing to a constant voltage pulse induced in the pick-up coil, the sensor has been used for rotation sensing and other applications [9,10]. Recently, the Wiegand sensor has attracted significant attention as a power supply for the battery-less operation of electric devices and for energy harvesting [11]. The rotation or vibration of even a single magnet is capable of generating an alternating magnetic field to yield consecutive pulse voltages [11,12]. We found that the pulse voltage is obtained from a wire excited by only 1–2-mm-long vibrations of a small magnet [12]. Although the Wiegand sensor is used industrially as an energy harvester for battery-less rotary encoders [13], there have been few research reports on either its basics or its applications [14,15,16].

The motivation of this work was to determine an equivalent circuit for the Wiegand sensor. The circuit design and simulation are essential for developing applications which use Wiegand sensors. The advantage of using a Wiegand sensor as an energy-harvesting element is that it can generate electrical power even from extremely slow movement of a magnet [12], which may not be achieved by other vibration-type energy harvesters. We found that a single Wiegand pulse generates an electrical power of 600 nJ [11]. In this paper, we demonstrate the concept of an equivalent circuit for the Wiegand sensor, which is expressed by the intrinsic pulse voltage (time-depending waveform), internal resistance, and inductance of the pick-up coil. The circuit simulation using this equivalent circuit gives the output characteristics of the Wiegand sensor, which agrees with the experimental results.

## 2. Wiegand Sensor

### 2.1. Pulse Voltage from the Wiegand Sensor

**Figure 1:**
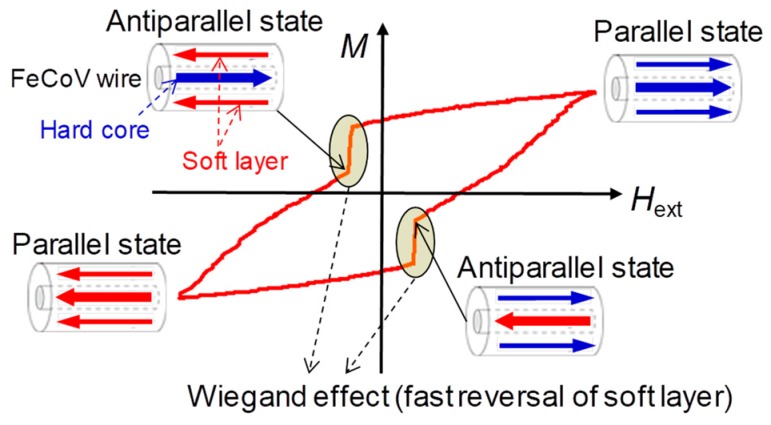
Schematics of magnetization curve and magnetization states of the Wiegand wire.

In our research, a twisted magnetic wire composed of Fe_0.4_Co_0.5_V_0.1_ was used [11,12], with a length and diameter as 11 mm and 0.25 mm, respectively. The wire has a two-layer magnetic structure, as illustrated in Figure 1. The outer layer and inner core exhibit magnetically soft and hard properties with their coercive forces at 1.6 kA/m and 6.4 kA/m, respectively. In order to achieve this specific magnetic property of the wire, annealing and torsion stress are applied to the wire. The magnetic properties of twisted FeCoV wires, depending on the conditions of annealing and torsion stress, have been previously reported in detail [6]. A torsion stress is first applied to the wire during its preparation. After the stress is released, the outer layer near the surface becomes magnetically soft, and the inner core remains magnetically hard. The wire exhibits a uniaxial magnetic anisotropy in the longitudinal direction. Because of this magnetic structure, magnetizations of the two layers are parallel or antiparallel to each other, depending on the intensity of the applied external magnetic field *H*_ex_, as shown in Figure 1.

**Figure 2:**
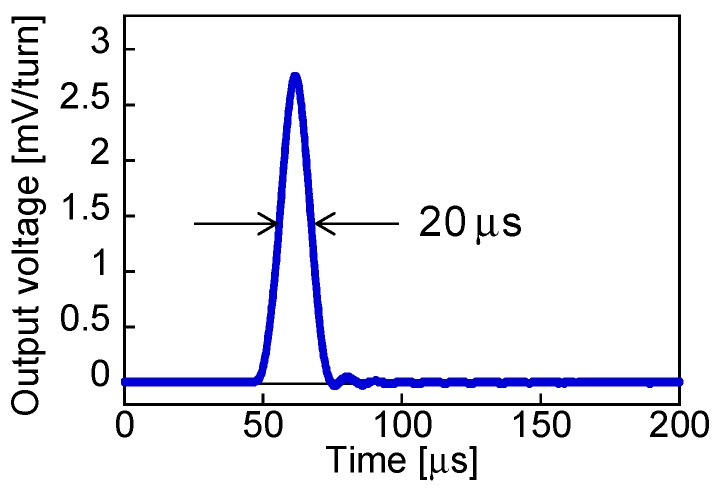
Example of a waveform of pulse voltage obtained from the Wiegand sensor.

When the magnetization of the soft layer was reversed, accompanied with a large Barkhausen jump, the pulse voltage was induced in the pick-up coil, as shown in Figure 2. After this abrupt change in magnetization of the soft layer from parallel to antiparallel states to that of the hard core as shown in Figure 1, the magnetostatic energy of the wire is reduced. The number of turns of the coil used in this study was 3000. The peak voltage was 2.5–3.0 V per one turn of the pick-up coil. The domain wall in the FeCoV wire was reported to be moving at approximately 500 m/s, which is independent of the change rate of the applied field intensity [17]. Owing to the fast movement of the domain, the full width of the induced pulse was 20 μs.

### 2.2. Equivalent Electrical Circuit for the Wiegand Sensor

**Figure 3:**
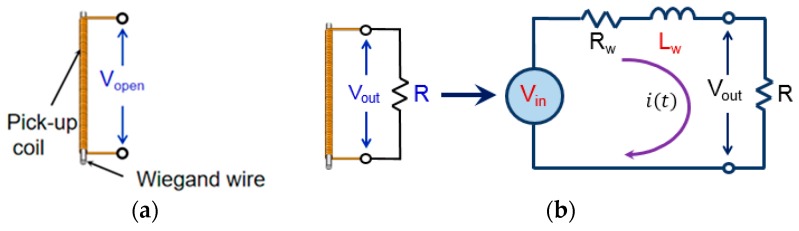
(**a**) Open-circuit voltage and (**b**) equivalent electrical circuit model of the Wiegand sensor.

**Figure 4:**
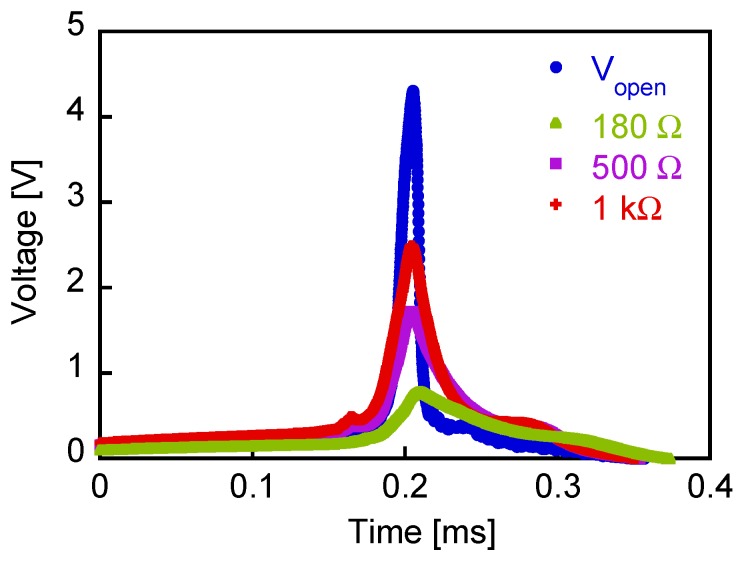
Experimentally measured waveforms of the open-circuit voltage and output voltages.

The output voltage shown in Figure 2 is the open-circuit voltage, *V*_open_, induced across both the ends of the pick-up coil, as shown in Figure 3a. In practical applications, the Wiegand sensor is connected to a load or other electrical circuits. The output voltage, *V*_out_, across the load resistance indicated in Figure 3b was measured. An alternating magnetic field of 3.2 kA/m in amplitude and at a frequency of 1 kHz was applied to the wire in order to switch the soft layer of the wire continuously. Figure 4 shows the typical waveforms of *V*_out_. The load resistance of *R* = 180 Ω, 500 Ω, or 1 kΩ was connected to the Wiegand sensor. *V*_out_ is smaller than *V*_open_ because of the voltage drop due to the current flow.

In order to evaluate and develop the electrical circuits used in applications of the Wiegand sensor, an equivalent circuit model is proposed in this paper. The Wiegand sensor consisted of a Wiegand wire and a pick-up coil, which are shown in the equivalent electrical circuit in Figure 3b, where *V*_in_, *R*_w_, and *L*_w_ are the intrinsic pulse voltage as the power source, internal resistance, and internal inductance of the pick-up coil in the Wiegand sensor, respectively. The output pulse voltage depending on the load resistance, *R*, can be explained by the equivalent electrical circuit shown in Figure 3b.

## 3. Experimental Method

### 3.1. Determination of the Circuit Parameters for the Wiegand Sensor

The internal resistance, *R*_w_, and inductance, *L*_w_, were measured using an LCR meter (HIOKI IM3536). *R*_w_ = 180 Ω was obtained, which agrees with the value determined by the simple current-voltage measurement of the pick-up coil in the Wiegand sensor. *L*_w_ = 2.54 mH was obtained by the LCR measurement, but the inductance depends on the magnetization state of the magnetic wire in the sensor [18]. Therefore, it is not appropriate to use the inductance value measured by an LCR meter. In order to evaluate an electrical circuit connected to the Wiegand sensor, the effective value of *L*_w_, which gives the correct circuit simulation, is significant.

In order to determine the effective value of *L*_w_ and the time-dependent intrinsic pulse voltage, *V*_in_, circuit simulation using MATLAB^®^/Simulink^®^ was performed.

In the circuit shown in Figure 3b, the following equations are derived:

(1) $$V_{in}=\left(R+R_{w}\right)i\left(t\right)+L_{w}\frac{di\left(t\right)}{dt}$$

(2) $$V_{out}=i\left(t\right)R$$

where *i*(t) and *V*_out_ are the current in the circuit and voltage across the load resistance *R*, respectively. These equations are converted by the Laplace transformation to:

(3) $$V_{out}=\frac{R_{w}}{L_{w}\;s+\left(R+R_{w}\right)}V_{in}$$

**Figure 5:**
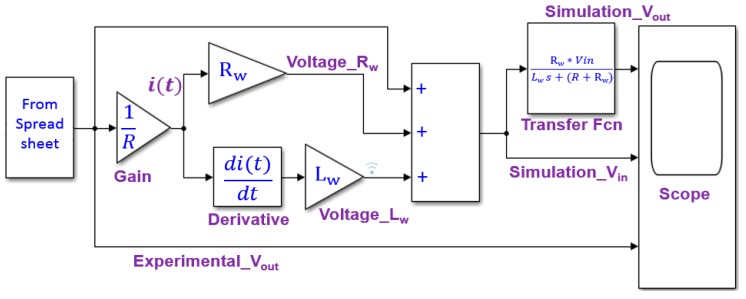
Block diagram for simulating the electrical circuit model of the Wiegand sensor by MATLAB/Simulink.

Figure 5 shows the block diagram for the electrical circuit model. By using the experimentally measured values of *R*_w_ and *V*_out_, *L*_w_ and *V*_in_ were determined by simulation. “From Spreadsheet block” gives the input data by referring to the measured waveform of *V*_out_, “Gain block” gives the circuit current *i*(*t*), and “Derivative block” calculates di(t)/dt. After assuming the internal inductance *L*_w_, the intrinsic pulse voltage *V*_in_ is calculated. By using this simulated result of *V*_in_ and “Transfer Fcn block” by processing Equation (3), the waveform of *V*_out_ is obtained. By fitting the calculated and experimentally measured waveform of *V*_out_, *L*_w_ and the waveform of *V*_in_ can be determined.

### 3.2. Full-Wave Bridge Rectifier Circuit for Wiegand Pulse Voltage

**Figure 6:**
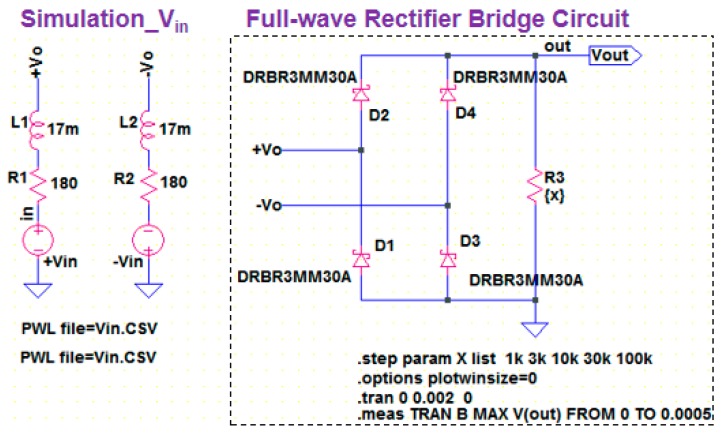
Simulation of full-wave bridge rectifier circuit connected to the Wiegand sensor by LTspice.

In the practical application of the Wiegand sensor for power generation and energy harvesting, a bridge rectifier circuit is normally used. The rectifier circuit prevents the backflow current to the Wiegand sensor, which is a conductive element composed of the inductor and resistor as well as a power source. The circuit response of the Wiegand sensor connected through a full-wave bridge rectifier circuit was studied. We measured the output voltage across the load resistor through a full-wave bridge rectifier circuit connected with the Wiegand sensor, and also calculated the voltage using LTspice^®^. As shown in Figure 6, the Wiegand sensor as a power source is expressed by *V*_in_, *R*_w_, and *L*_w_, which are determined by the MATLAB/Simulink simulation. When we apply an alternating magnetic field to the Wiegand sensor, the magnetization of the magnetic wire is switched to positive and negative directions in one cycle of the applied field, resulting in positive and negative output pulse voltages. Therefore, +*V*_in_ and −*V*_in_ are used as input voltages to the full-wave bridge rectifier circuit. As a rectifying diode for the bridge circuit, a Schottky barrier diode, RBR3MM30A, commercially supplied by ROHM, was used. It is indicated as “DRBR3MM30A” in the figure, and “R3” in the figure is the load resistor.

## 4. Results and Discussion

### 4.1. Internal Inductance and Intrinsic Pulse Voltage of the Wiegand Sensor

**Figure 7:**
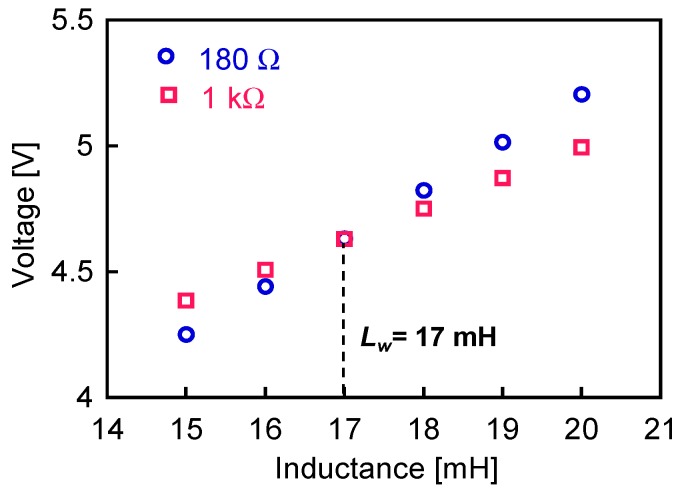
Peak values of intrinsic pulse voltage, *V*_in_, as functions of internal inductances of the Wiegand sensor. Load resistor *R* was either 180 Ω or 1 kΩ.

Figure 7 shows the peak values of the intrinsic pulse voltage, *V*_in_, calculated as functions of the internal inductance, *L*_w_. The load resistance used was either *R* = 180 Ω or *R* = 1 kΩ. Ideally, *V*_in_ should be constant and independent of *R*; thus, we chose the inductance value at which the intrinsic pulse voltage was most similar for the two load resistances. From the figure, *L*_w_ is thus determined to be 17 mH. This is an effective value of the inductance for circuit response.

**Figure 8:**
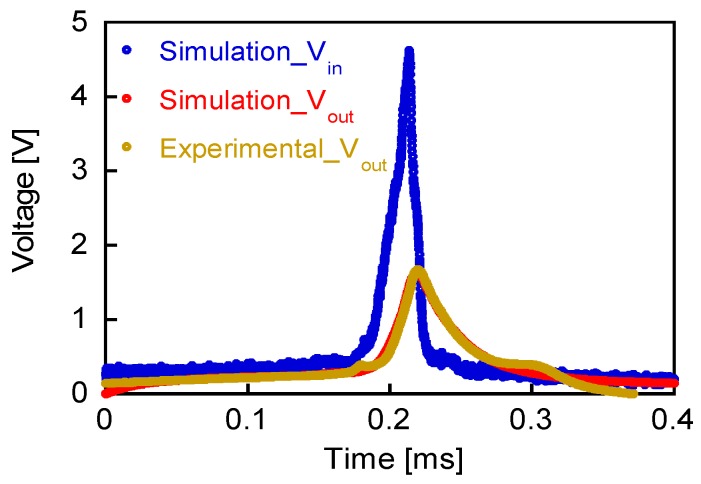
Waveforms of calculated *V*_in_, *V*_out_, and experimentally measured *V*_out_.

Figure 8 shows the waveforms of the calculated *V*_in_, *V*_out_, and experimentally measured *V*_out_ (*L*_w_ = 17 mH and *R* = 500 Ω). The calculated and measured waveforms of *V*_out_ agree with each other, and the simulation is thus accurately processed. The waveform of the intrinsic pulse voltage of the Wiegand sensor, *V*_in_, is therefore noted to be correctly determined with the abovementioned component values. The peak value of *V_in_* for the pick-up coil of 3000 turn is 4.62 V.

### 4.2. Output Voltage through Full-WAVE Bridge Rectifier Circuit

**Figure 9:**
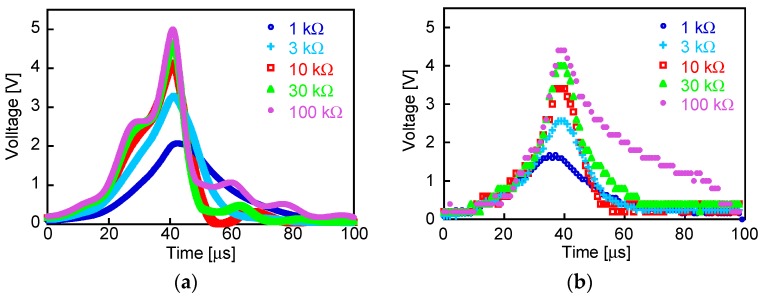
Waveforms of (**a**) experimentally measured and (**b**) calculated output voltage, *V*_out_, across the load resistor. Load resistance *R* = 1, 3, 10, 30, and 100 kΩ.

Figure 9a shows the waveforms of the experimentally measured output voltage, *V*_out_. The load resistance *R* = 1, 3, 10, 30, or 100 kΩ. In the experiment, the waveforms are superimposed with several additional pulse signals, which are possibly resonant signals due to the inductance of the Wiegand sensor and the internal capacitances of the Schottky barrier diodes.

**Figure 10:**
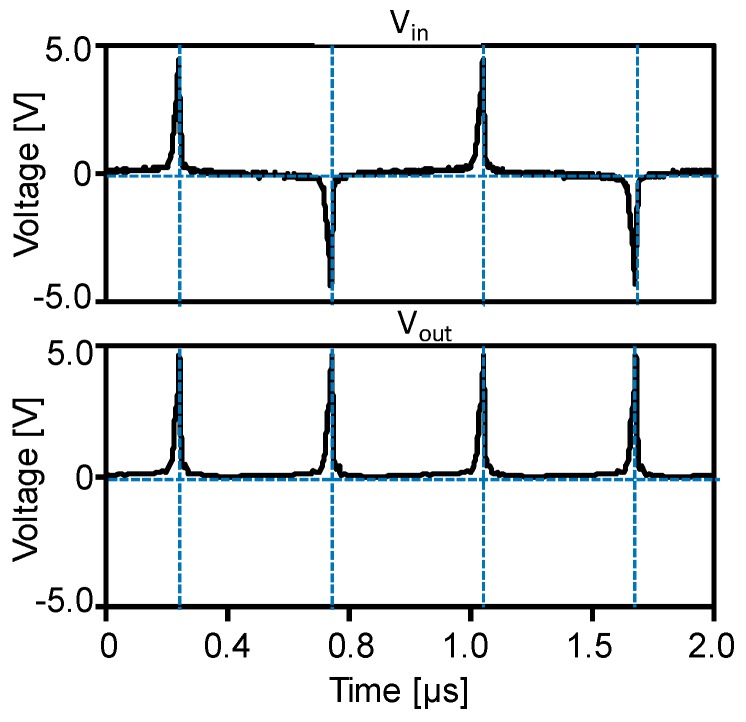
Waveforms of intrinsic pulse voltage, *V*_in_, from the Wiegand sensor calculated using MATLAB/Simulink (top), and output voltage across load resistor *R* = 10 kΩ, *V*_out_, calculated using LTspice (bottom).

The upper graph of Figure 10 shows the waveform of the intrinsic pulse voltage, *V*_in_ as the power source of the Wiegand sensor calculated using MATLAB/Simulink. It is assumed that an alternating magnetic field of 3.2 kA/m amplitude and 1 kHz frequency was applied to the circuit. As the magnetization of the soft layer switches along both directions of the wire, alternating positive and negative pulses of period 1 ms are observed. The bottom graph of Figure 10 shows the waveform of the output voltage across the load resistor of 10 kΩ, *V*_out_, calculated using LTspice. Figure 10 shows the waveforms of the calculated output voltage, *V*_out_, across the load resistor, *R*. The experimental and calculated waveforms shown in Figure 9a,b are observed to be similar, implying that whole process of the simulation including determination of *V*_in_ and *L*_w_ is appropriate. The optimized value of *L*_w_ = 17 mH is quite different from the 2.54 mH obtained by the LCR measurement; however, it is useful as the effective inductance for reproducing the experimental results by circuit simulation. The calculated waveforms do not exhibit superimposed additional pulse signals because the LTspice simulation does not consider the internal capacitances of the Schottky barrier diodes. In the simulation, the power loss at the bridge rectifier circuit is considered, which is 26 nJ for a single Wiegand pulse. Although this loss is non-negligible compared to the power consumption at the load resistor of 57 nJ, the rectifying circuit is necessary in order to prevent the backflow current to the Wiegand sensor, as discussed in Section 3.2.

**Figure 11:**
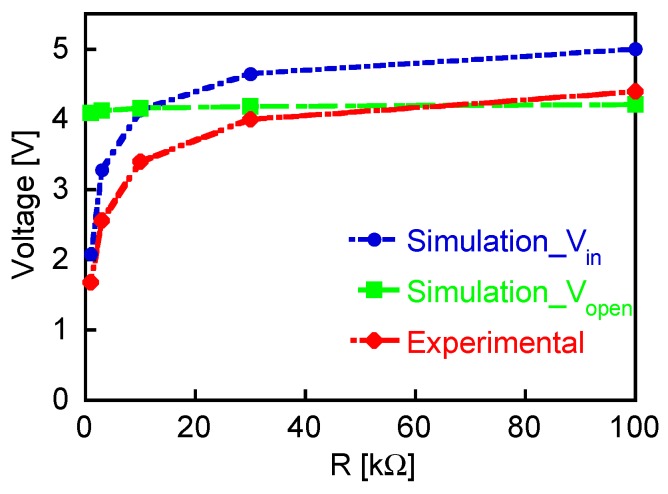
Peak values of experimentally measured and calculated output pulse voltages, V_out_, plotted as functions of load resistance, *R*. For simulation, (1) *V*_in_ derived using MATLAB/Simulink with considered *R*_w_ and *L*_w_, or (2) measured open-voltage, *V*_open_, is used as the power source of the Wiegand sensor.

Figure 11 shows the peak values of the experimentally measured and calculated output pulse voltages plotted as functions of the load resistances. In the calculation, *V*_in_, derived using MATLAB/Simulink with the considered *R*_w_ and *L*_w_, is used as the power source of the Wiegand sensor. Their dependences on *R* are quite similar. The reason for the difference of ~0.5 V between the experiment and simulation results is not clear. Even with this difference, simulations that use the open-circuit voltage, *V*_open_, cannot reproduce the experimental results. We can thus successfully demonstrate the effectiveness of introducing the intrinsic pulse voltage, *V*_in_, as well as internal resistance and inductance of the Wiegand sensor. These circuit parameters were derived from the experimental results measured at room temperature. Both the open-circuit and intrinsic voltages are dependent on the temperature because the magnetization of the wire reduces with increasing temperature. The inductance *L*_w_ is also dependent on the magnetization. In case of simulating a circuit operated at temperatures other than the room temperature, it is necessary to perform another measurement at that temperature and to calculate the circuit parameters.

## 5. Conclusions

In this study, we propose the concept of an intrinsic pulse voltage from the Wiegand sensor as its power source. Conventionally, the pulse voltage has been discussed as the measured open-circuit voltage across the pick-up coil of the sensor. For the analysis of a circuit connected to the Wiegand sensor, this open-circuit voltage is not applicable because of the voltage drop due to current flow. Using MATLAB/Simulink simulations, we determined the circuit parameters of the internal resistance and inductance of the pick-up coil, and the time-dependent (waveform of the) pulse voltage as the intrinsic pulse voltage. As an example of the circuit simulation, circuit responses through the full-wave bridge rectifier circuit connected to the Wiegand sensor were calculated. The calculated output voltages agree with the experimentally measured values, and the effectiveness of using the intrinsic pulse voltage, and the internal resistance and inductance of the Wiegand sensor, is thus demonstrated.
